# Homoarginine—A prognostic indicator in adolescents and adults with complex congenital heart disease?

**DOI:** 10.1371/journal.pone.0184333

**Published:** 2017-09-08

**Authors:** Tanja Raedle-Hurst, Marieke Mueller, Andreas Meinitzer, Winfried Maerz, Thomas Dschietzig

**Affiliations:** 1 Department of Pediatric Cardiology, Saarland University Medical Center, Homburg/Saar, Germany; 2 Clinical Institute of Medical and Chemical Laboratory Diagnostics, Medical University of Graz, Graz, Austria; 3 Medical Clinic V (Nephrology, Hypertensiology, Rheumatology, Endocrinology, Diabetology), Medical Faculty Mannheim, University of Heidelberg, Mannheim, Germany; 4 Synlab Academy, Synlab Holding Deutschland GmbH, Mannheim and Augsburg, Germany; 5 Charité – University Medicine Berlin, Medical Clinic for Cardiology and Angiology, Campus Mitte, Berlin, Germany; 6 Immundiagnostik GmbH, Bensheim, Germany; Kurume University School of Medicine, JAPAN

## Abstract

**Background:**

Homoarginine (hArg) has been shown to be of prognostic value in patients with chronic left heart failure. The present study aims to assess the clinical utility and prognostic value of hArg levels in patients with complex congenital heart disease (CHD).

**Methods:**

Plasma hArg levels were measured in 143 patients with complex CHD and compared to clinical status, echocardiographic and laboratory parameters as well as the occurrence of adverse cardiac events.

**Results:**

Median hArg levels were 1.5 μmol/l in CHD patients as compared to 1.70 μmol/l in healthy controls (p = 0.051). Median hArg levels were lowest in patients with Fontan palliation (1.27 μmol/l) and Eisenmenger physiology (0.99 μmol/l) and decreased with the severity of adverse cardiac events with lowest values found in patients prior to death or overt heart failure (0.89 μmol/l). According to ROC analysis, the most important predictors of adverse cardiac events were hArg levels (AUC 0.837, p<0.001, CI 0.726–0.947), NYHA class (AUC 0.800, p<0.001, CI 0.672–0.928) and NT-proBNP levels (AUC 0.780, p<0.001, CI 0.669–0.891). The occurrence of overt heart failure or death due to progressive heart failure were best predicted by NYHA class (AUC 0.945, p<0.001, CI 0.898–0.992), hArg levels (AUC 0.911, p<0.001, CI 0.850–0.971) and NT-proBNP levels (AUC 0.877, p<0.001, CI 0.791–0.962), respectively.

**Conclusion:**

In patients with complex CHD, hArg levels can predict adverse cardiac events as reliably as or even better than NT-proBNP levels and thus might be of prognostic value in this subset of patients.

## Introduction

Natriuretic peptides (NPs) such as BNP or NT-proBNP are known to be of diagnostic and prognostic value in patients with congenital heart disease (CHD) [1–3]. Risk stratification using NP levels in this subset of patients, however, may be challenging due to the heterogeneity of malformations, the different pathophysiology (biventricular versus univentricular physiology) and the presence of hypoxia (decreased oxygen saturation) [4,5]. Moreover, confounders such as age, sex, renal dysfunction and obesity may further influence NP levels [6–8]. Since NP are mainly secreted from the left ventricle due to increasing myocardial wall stress and thus are “load-dependent”, medication also may alter NP levels. In CHD patients, the pathophysiology of the underlying malformation plays an important role in the assessment of NP levels. Patients with a systemic morphological right ventricle or univentricular physiology after the Fontan procedure usually show lower NP levels than patients with biventricular physiology. Nevertheless, BNP has been proven to predict mortality in adults with CHD [9].

Homoarginine (hArg) is an amino acid that plays an important role in nitric oxide (NO) metabolism increasing the availability of NO by inhibiting the enzyme arginase to increase L-arginine for NO production [10,11]. hArg itself may also serve as substrate for NO synthesis. It has been shown that lack of NO is associated with endothelial as well as myocardial dysfunction [12,13]. There are two sources of homoarginine: a) synthesis from lysine in an “alternative urea cycle” b) amidation of lysine by L-arginine:Glycine amidinotransferase [14]. Although the mechanisms linking hArg to cardiovascular disease are not completely understood, previous studies in cardiovascular high risk patients as well as in patients with chronic left heart failure demonstrated that low hArg levels are associated with adverse cardiovascular outcomes and all-cause mortality [14–16]. These results suggest that hArg may be an independent prognostic marker in patients with cardiovascular disease and/or left heart failure.

To our knowledge, studies investigating hArg levels in CHD patients are lacking. However, serum levels of asymmetrical dimethylarginine, a potent endogenous inhibitor of NO synthase competing with arginine for NO synthase binding, were measured in adults with CHD demonstrating to be more sensitive than NT-proBNP in detecting different stages of heart failure in this cohort of patients [17]. Moreover, symmetrical dimethylarginine was found to be superior to NT-proBNP in detecting systemic ventricular dysfunction in CHD patients after atrial repair for transposition of the great arteries [18]. Both studies demonstrate the potential diagnostic value of these markers, however, prognostic utility hasn’t been addressed.

The aim of the present study therefore was to analyse hArg levels in patients with complex CHD in order to assess its clinical utility and prognostic value in this subset of patients.

## Materials and methods

### Patients

155 patients with various types of complex CHD were screened consecutively in our CHD outpatient clinic between 22/12/2008 and 27/7/2015 to participate in the study. Complex CHD was defined as CHD of mostly great complexity according to ACC/AHA guidelines [19] thus including patients after corrective surgery of congenital right heart disease (CRHD) such as pulmonary atresia or tetralogy of Fallot, patients with a systemic morphological right ventricle (SRV), patients with single ventricle physiology after Fontan palliation (FONT) and patients with a non-corrected cyanotic heart defect or Eisenmenger physiology (EIS). All other patients with CHD of simple or moderate complexity were not approached for participation. 12/155 patients with complex CHD could not be included in the analysis due to various reasons (3 patients were lost to follow-up, 2 patients refused to participate, 7 patients had missing relevant laboratory or echocardiographic parameters) giving a drop-out rate of 7.7%.

Finally, a total of 143 patients with complex CHD were enrolled in the study. Mean age was 27.5 ± 12.0 years (range 11–73 years). 64 patients were female and 79 male. 49/143 (34.3%) patients had surgically corrected CRHD, 35/143 (24.5%) patients a SRV of whom 10 patients had congenitally corrected transposition of the great arteries and 25 patients d-transposition of the great arteries after atrial switch operation, 43/143 (30.1%) patients a single ventricle physiology with Fontan palliation (FONT) and 16/143 (11.2%) patients a non-corrected cyanotic heart defect or Eisenmenger physiology (EIS) (S1 Dataset). Follow-up investigations were made with special emphasis on the occurrence of adverse cardiac events such as clinical signs of overt heart failure including pleural effusions, ascites or significant peripheral edema with weight gain as well as the occurrence of supraventricular arrhythmias or atrial fibrillation. At each follow-up visit, a structured history and physical exam, a 12-lead surface electrocardiogram, measurement of blood pressure and transcutaneous oxygen saturation at rest and conventional two-dimensional echocardiography were performed in all patients. Moreover, venous blood was drawn for routine laboratory tests and blood sampling in all patients after echocardiography. Blood samples of patients were compared to those of 26 healthy subjects with a mean age of 30.8 ± 15.3 years (range 12–61 years) (S1 Dataset). The study complies with the Declaration of Helsinki and was approved by the local ethics committee, specifically the Saarland medical association ethical board. All participants or their guardians gave written and informed consent before enrolment.

### Conventional two-dimensional echocardiography

Two-dimensional echocardiography was performed using a Vivid 9 ultrasound system (GE Healthcare, Horten, Norway) with a transducer operating at 3.5 MHz. Apical 4-chamber views were acquired for the planimetric calculation of the ejection fraction (EF) as well as endsystolic and enddiastolic volumes of the systemic ventricle according to the modified Simpson’s method [20]. Pulsed-wave Doppler echocardiography was performed to obtain the outflow pattern above the aortic valve to measure velocity time integral (VTI). Continuous-wave Doppler was used to estimate pressure gradients across the aortic or pulmonary valve. Colored Doppler flow was used to assess the severity of regurgitation across the systemic atrioventricular and aortic valve.

### Biochemical analyses

Each venous blood sample was centrifuged and plasma removed, allocated and frozen at -80°C before analysis. NT-proBNP was analyzed using an electrochemiluminescence sandwich immunoassay (Cobas^®^ proBNP II, Roche Diagnostics, Basel, Switzerland) on the Elecsys^®^ 2010 analyzer. Levels of hArg were measured by high-performance liquid chromatography with solid phase extraction and precolumn derivatization as previously described [21]. Within-day coefficients of variation were 4.7% (1.21 μmol/L) and 2.2% (3.53 μmol/L). Liver function tests and creatinine measurements were performed using standard laboratory techniques. All biochemical analyses were performed by investigators blinded to the clinical data of the patients.

### Data analysis

Clinical data of the patients were collected from medical records. The two-dimensional echocardiographic and Doppler images were stored digitally and analysed on an Echopac server (Echopac Version 6.1.0, GE Healthcare). Echocardiographic data sets were assessed by investigators blinded to the laboratory results.

Patients with adverse cardiac events were categorized into 3 subgroups: (1) patients with sporadic new-onset supraventricular arrhythmias, (2) patients with recurrent supraventricular arrhythmias or permanent atrial fibrillation and (3) patients who died or presented with overt heart failure including failure of the Fontan circuit during follow-up.

### Statistical analysis

Data were analysed using standard statistical software (SPSS version 19; SPSS Inc., Chicago, Illinois). Continuous variables are expressed as mean ± standard deviation or median and interquartile range as appropriate. Differences between unpaired groups were analysed using a Mann-Whitney-U test for continuous variables and a chi-square test (or Fisher exact test, if numbers were small) for nominal variables. Correlations were evaluated using Spearman’s regression coefficient. Differences between paired groups were analysed using a Wilcoxon-signed-rank test for continuous variables. Receiver-operating characteristic curve (ROC) analysis was used in a univariate approach for the prediction of adverse cardiac events. A two-tailed p-value <0.05 was considered statistically significant.

## Results and discussion

### Results

#### Clinical, echocardiographic and routine laboratory data

During follow-up, a total of 33 adverse cardiac events occurred in 30/143 (21.0%) patients: 4 patients died (1 patient due to sudden cardiac death, 3 patients due to progressive heart failure), 15 patients presented with overt heart failure or a failing Fontan circuit, 6 patients had sporadic new-onset supraventricular arrhythmias, 8 patients had recurrent supraventricular arrhythmias or permanent atrial fibrillation indicating high atrial arrhythmic burden. Of the 30 patients, 4 had CRHD (13.3%), 8 SRV (26.7%), 12 FONT (40%) and 6 EIS (20%) as underlying CHD (S1 Dataset). Characteristics of patients with and without adverse cardiac events are shown in Table 1.

**Table 1:** Characteristics of patients with and without adverse cardiac events^#^.

Variables	All patients	Patients without adverse cardiac events	Patients with adverse cardiac events	p-value*
	(n = 143)	(n = 113)	(n = 30)	
Age at follow-up (years)	27.5 ± 12.0	26.5 ± 10.8	31.1 ± 15.5	ns
Number of patients with Fontan palliation or Eisenmenger physiology	59/143 (41%)	41/113 (36.3%)	18/30 (60%)	0.02
NYHA functional class	1.6 ± 0.7	1.4 ± 0.5	2.3 ± 0.9	< 0.001
Systolic blood pressure (mmHg)	121.4 ± 14.7	121.9 ± 13.6	119.9 ± 18.3	ns
Diastolic blood pressure (mmHg)	70.8 ± 9.2	71.2 ± 9.2	69.1 ± 9.2	ns
Transcutaneous oxygen saturation at rest (%)	94.5 ± 5.8	95.5 ± 4.4	90.5 ± 8.4	< 0.001
Ejection fraction of SV (%)	50.9 ± 11.3	53.0 ± 9.2	43.0 ± 14.9	< 0.001
Enddiastolic volume of SV (ml)	110.0 ± 43.7	106.3 ± 39.9	123.8 ± 54.4	ns
Endsystolic volume of SV (ml)	55.8 ± 32.0	50.9 ± 24.4	74.3 ± 47.6	0.004
VTI above aortic valve (cm)	24.0 ± 5.0	24.7 ± 4.5	21.4 ± 5.9	0.004
Creatinine (mg/dl)	0.81	0.80	0.84	ns
	(0.70–0.95)	(0.70–0.94)	(0.59–1.1)	
Estimated GFR (ml/min)	102.1	103.4	96.3	ns
	(90.4–118.9)	(92.6–119.6)	(82.7–118.3)	
γGT (U/l)	41.0	34.0	72.0	< 0.001
	(24.0–71.0)	(22.0–61.0)	(55.5–102.3)	
Albumin (g/l)	47.0	47.0	45.0	0.034
	(44.0–49.0)	(45.0–49.0)	(40.8–50.0)	
NT-proBNP (pg/ml)	164.7	136.7	651.0	< 0.001
	(77.4–501.1)	(68.9–275.8)	(275.1–1326.2)	
Homoarginine (μmol/l)	1.5	1.63	1.03	< 0.001
	(1.12–1.98)	(1.28–2.12)	(0.78–1.36)	

CHD, congenital heart disease; NYHA, New York Heart Association; SV, systemic ventricle; VTI, velocity time integral; GFR, glomerular filtration rate

^#^ Mean ± standard deviation or median (interquartile range) are used

* Patients with compared to those without adverse cardiac events

In patients with adverse cardiac events, NYHA class was significantly higher, mean EF of the systemic ventricle and VTI above the aortic valve were significantly lower as compared to patients without any cardiac event during follow-up. Furthermore, concentrations of γGT and NT-proBNP were significantly elevated whereas hArg levels were significantly reduced in patients with adverse cardiac events. Median creatinine levels and estimated glomerular filtration rate were normal in both groups indicating no relevant renal dysfunction.

#### Homoarginine concentrations

**Fig 1:**
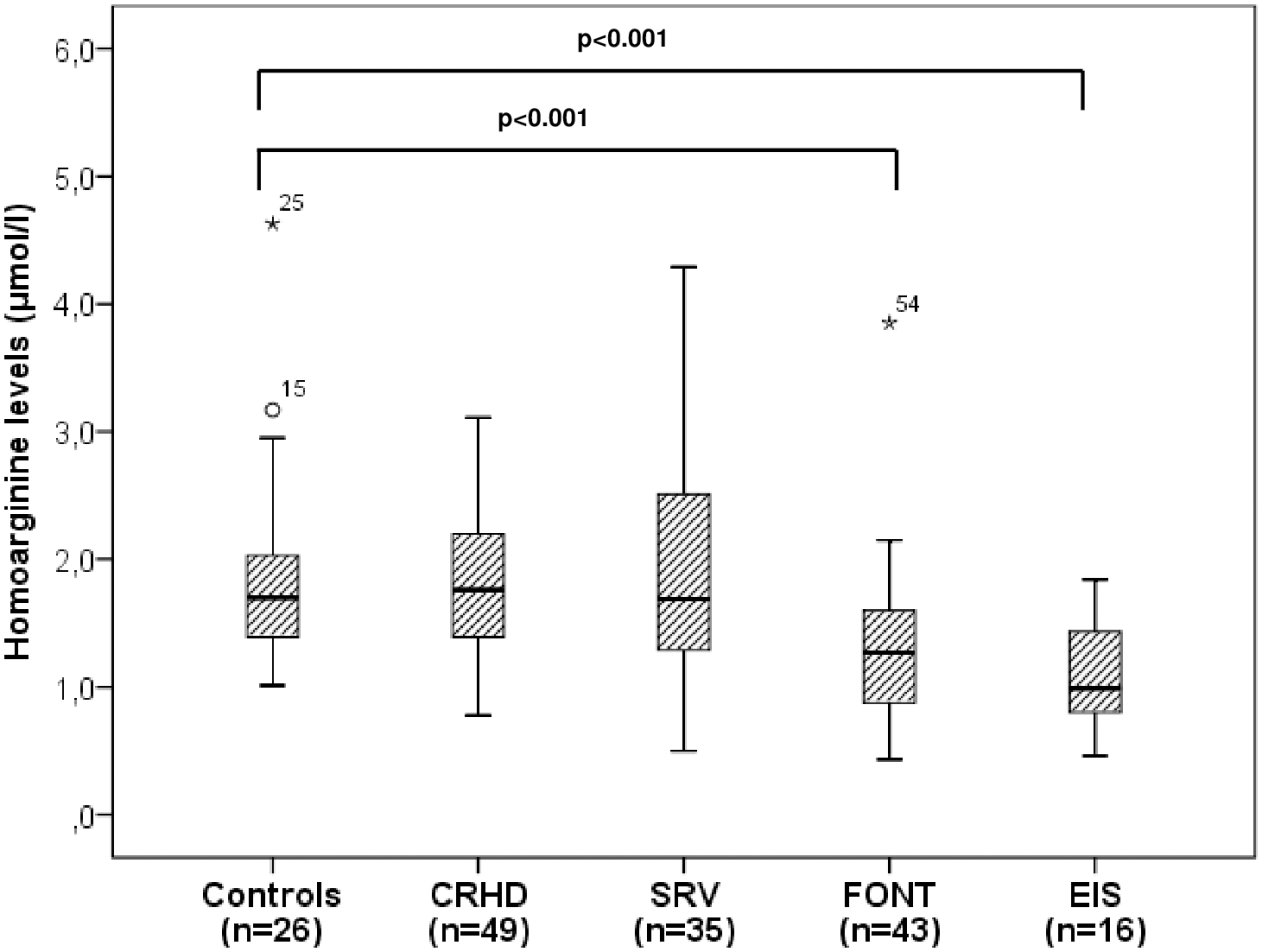
Boxplots displaying homoarginine levels in healthy controls and patients with various types of complex heart defects. CRHD, corrected congenital right heart disease; SRV, systemic right ventricle; FONT, Fontan palliation; EIS, Eisenmenger physiology or unrepaired cyanotic heart defect.

Homoarginine levels ranged from 0.43 to 4.29 μmol/l with a median of 1.5 μmol/l (1.12–1.98 μmol/l) in the patient group. In general, median hArg levels were reduced in patients compared to healthy controls showing a median hArg level of 1.70 μmol/l (1.39–2.08 μmol/l; p = 0.051). A marked and significant reduction of hArg levels, however, was seen in patients after Fontan palliation with a median of 1.27 μmol/l (0.84–1.6 μmol/l) and in those with Eisenmenger physiology with a median of 0.99 μmol/l (0.79–1.47 μmol/l) (p<0.001) (Fig 1) (S1 Dataset).

**Fig 2:**
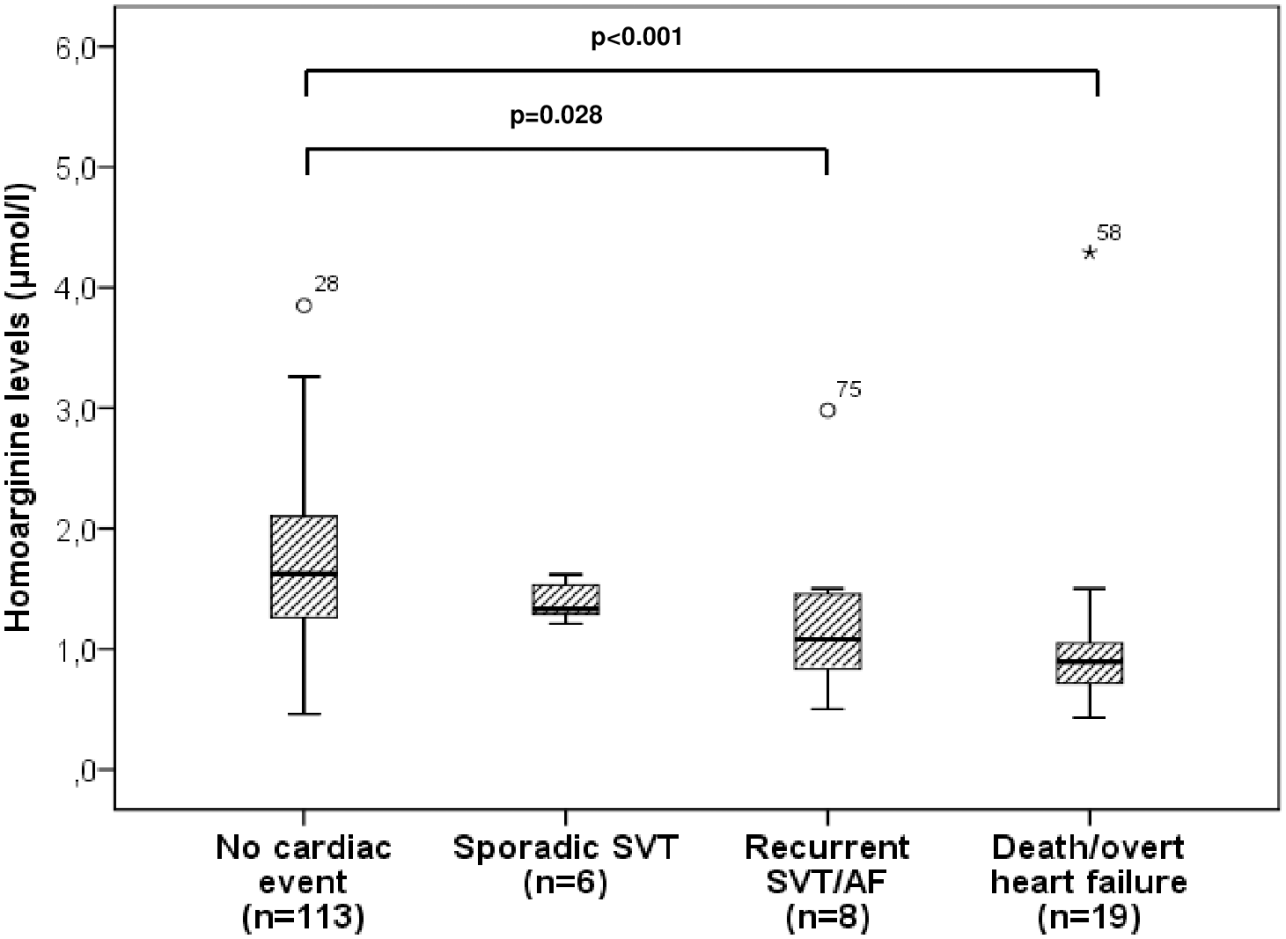
Boxplots demonstrating homoarginine levels in patients with different adverse cardiac events. SVT, supraventricular tachycardia; AF, atrial fibrillation.

Median hArg levels were also significantly reduced in patients with adverse cardiac events compared to patients without (p<0.001) (Table 1). Moreover, differences in hArg levels were found corresponding to the severity of adverse cardiac events with the lowest hArg levels found in patients prior to death or overt heart failure (Fig 2).

Levels of hArg were weakly related to NT-proBNP levels (r = -0.285; p = 0.001). Relations of both biomarkers to clinical, echocardiographic and laboratory parameters are given in Table 2.

**Table 2:** Relation of hArg and NT-proBNP with different variables*.

	hArg		NT-proBNP	
	r	p-value	r	p-value
Age at follow-up	0.090	ns	0.488	< 0.001
NYHA functional class	-0.294	< 0.001	0.551	< 0.001
Systolic blood pressure	0.194	0.020	-0.052	ns
Diastolic blood pressure	0.274	0.001	-0.019	ns
Transcutaneous oxygen saturation at rest	0.487	< 0.001	-0.212	0.011
Ejection fraction of SV	0.075	ns	-0.559	< 0.001
Enddiastolic volume of SV	-0.035	ns	0.083	ns
Endsystolic volume of SV	-0.035	ns	0.303	< 0.001
VTI above aortic valve	0.152	ns	-0.293	< 0.001
Creatinine	-0.011	ns	0.134	ns
Glomerular filtration rate	0.069	ns	-0.288	0.001
Albumin	0.155	ns	-0.223	0.007
γGT	-0.306	< 0.001	0.274	0.001

hArg, homoarginine; NYHA, New York Heart Association; SV, systemic ventricle; VTI, velocity time integral

*Spearman rank correlation

NT-proBNP levels were significantly related to age at follow-up, NYHA functional class, trans-cutaneous oxygen saturation, ejection fraction and endsystolic volume of the systemic ventricle, VTI above the aortic valve, estimated glomerular filtration rate, albumin and γGT levels. In contrast, hArg levels were only significantly related to NYHA functional class, transcutaneous oxygen saturation and γGT levels but not to measures of systolic ventricular function or estimated glomerular filtration rate (S1 Dataset).

Repeated measurements of hArg and NT-proBNP concentrations were obtained in 41 of 143 patients of whom 8 patients presented with and 33 patients without adverse cardiac event. In the 8 patients with adverse cardiac event, median hArg levels decreased significantly from 1.43 μmol/l to 0.97 μmol/l prior to the event (p = 0.012) but also a significant increase in median NT-proBNP levels from 880.2 pg/ml to 1021.9 pg/ml was seen (p = 0.017). In the 33 patients without adverse cardiac event, no difference in median hArg or NT-proBNP levels was detected. However, in 11 of these 33 patients, a significant rise of NT-proBNP levels from a median baseline level of 381.1 pg/ml to a median level of 747.1 pg/ml (p = 0.003) was observed without acute or subsequent deterioration of the clinical status of the patients, clinical signs of overt heart failure, new onset arrhythmias or renal function changes. In contrast, hArg levels were stable in these patients and did not differ significantly (median baseline level of 1.77 μmol/l vs 1.78 μmol/l, p = 0.213).

#### Homoarginine and adverse cardiac events

**Fig 3:**
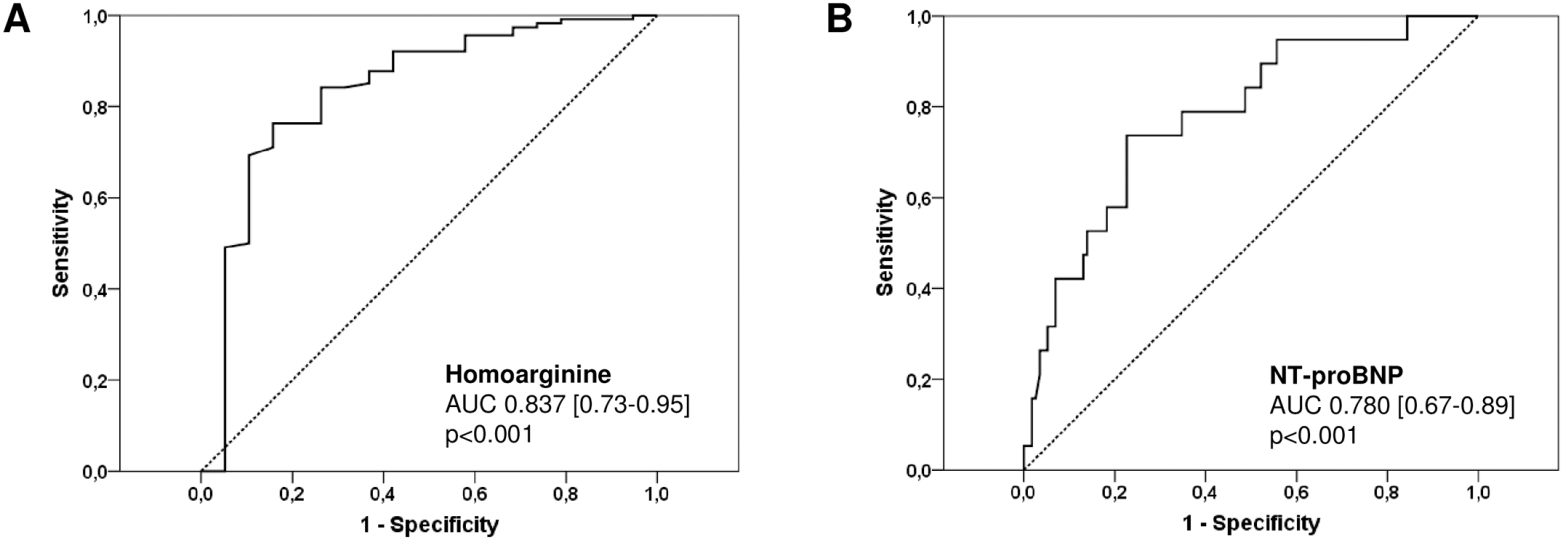
Receiver-operating characteristic (ROC) curves comparing sensitivity and specificity of hArg and NT-proBNP levels in predicting adverse cardiac events in general. AUC, area under the curve.

**Fig 4:**
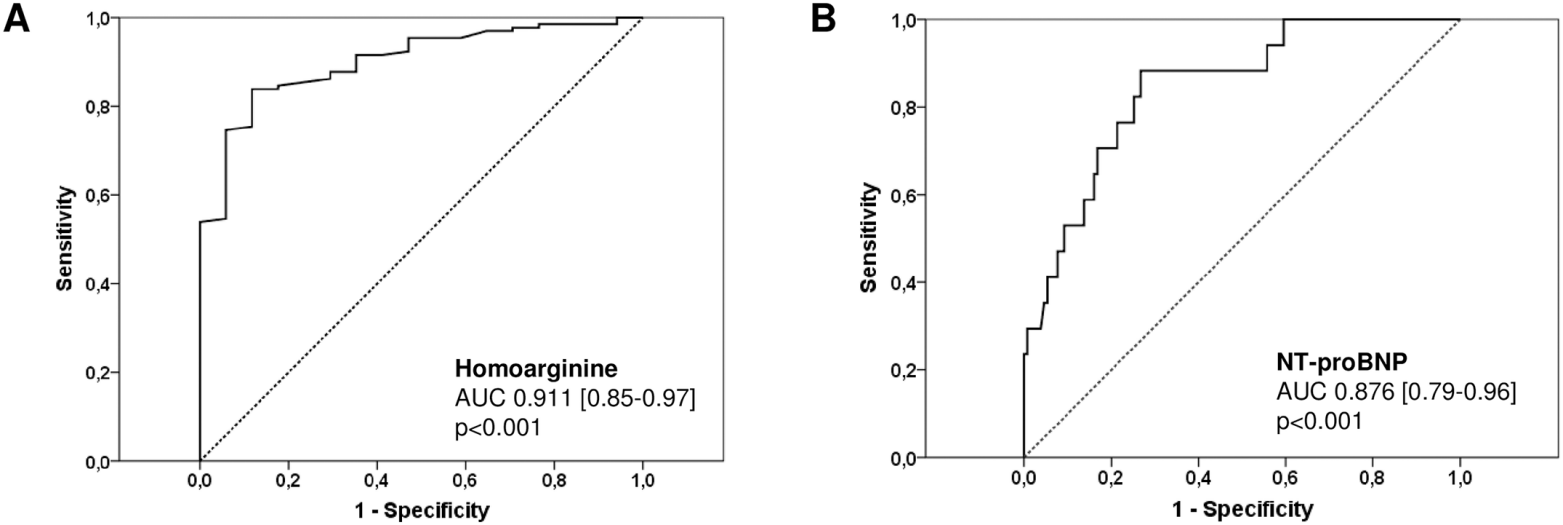
Receiver-operating characteristic (ROC) curves comparing sensitivity and specificity of hArg and NT-proBNP levels in predicting death due to progressive heart failure or occurrence of overt heart failure. AUC, area under the curve.

ROC curve analysis was used to identify predictors of adverse cardiac events in general (Fig 3) as well as predictors of death due to progressive heart failure or occurrence of overt heart failure in particular (Fig 4).

The most significant predictors of adverse cardiac events in patients with complex CHD were hArg levels (AUC 0.837, p<0.001, CI 0.726–0.947), γGT levels (AUC 0.804, p<0.001, CI 0.694–0.914), NYHA class (AUC 0.800, p<0.001, CI 0.672–0.928), NT-proBNP levels (AUC 0.780, p<0.001, CI 0.669–0.891), albumin levels (AUC 0.720, p = 0.005, CI 0.544–0.897) and ejection fraction of the systemic ventricle (AUC 0.667, p = 0.024, CI 0.507–0.826), respectively. The optimal cut-off of hArg for the prediction of adverse cardiac events in general was calculated to be 1.22 μmol/l with a sensitivity of 84.2%, specificity of 77.4%, positive predictive value of 38.1%, negative predictive value of 96.7% and overall accuracy of 78.4%. The optimal cut-off of NT-proBNP for the prediction of adverse cardiac events was calculated to be 341.2 pg/ml with a sensitivity of 73.7%, specificity of 77.4%, positive predictive value of 35.0%, negative predictive value of 94.7% and overall accuracy of 76.9%.

Death due to progressive heart failure or the occurrence of overt heart failure were best predicted by NYHA class (AUC 0.945, p<0.001, CI 0.898–0.992), hArg levels (AUC 0.911, p<0.001, CI 0.850–0.971), NT-proBNP levels (AUC 0.876, p<0.001, CI 0.791–0.962), albumin levels (AUC 0.743, p = 0.003, CI 0.551–0.935), γGT levels (AUC 0.732, p = 0.003, CI 0.579–0.885), EF of the systemic ventricle (AUC 0.723, p = 0.004, CI 0.560–0.886) and VTI above the aortic valve (AUC 0.720, p = 0.007, CI 0.550–0.889), respectively. The optimal cut-off of hArg for the prediction of death due to progressive heart failure or the occurrence of overt heart failure was calculated to be 1.08 μmol/l with a sensitivity of 93.8%, specificity of 85.3%, positive predictive value of 46.9%, negative predictive value of 99.0% and overall accuracy of 86.4%. The optimal cut-off of NT-proBNP was calculated to be 341.2 pg/ml with a sensitivity of 87.5%, specificity of 78.5%, positive predictive value of 35.9%, negative predictive value of 97.8% and overall accuracy of 79.6%. More detailed information is given in Table 3.

**Table 3:** Comparison of different parameters in predicting death due to progressive heart failure or the occurrence of overt heart failure.

Variables	Optimal cut-off value	Sensitivity	Specificity	PPV	NPV	Overall accuracy
Homoarginine	1.08 μmol/l	93.8%	85.3%	46.9%	99.0%	86.4%
NT-proBNP	341.2 pg/ml	87.5%	78.5%	35.9%	97.8%	79.6%
NYHA functional class	2.5	76.5%	96.6%	76.5%	96.6%	94.0%
Ejection fraction of SV	37.1%	50.0%	94.8%	57.1%	93.2%	89.3%
VTI above aortic valve	19.7 cm	57.1%	86.1%	33.3%	94.3%	83.0%
γGT	57.5 U/l	75.0%	71.9%	27.3%	95.3%	72.3%
Albumin	43.5 g/l	66.7%	87.5%	47.6%	93.9%	84.5%

PPV, positive predictive value; NPV, negative predictive value; NYHA, New York Heart Association; SV, systemic ventricle; VTI, velocity time integral

### Discussion

Recent studies have demonstrated the potential diagnostic and prognostic utility of hArg in patients with chronic left heart failure [15,22]. While measurements of symmetrical and asymmetrical dimethylarginine have been performed to assess their diagnostic utility in precisely defined subsets of CHD patients [17,18] this is, to our knowledge, the first study investigating hArg levels in a heterogeneous group of patients with complex CHD including analysis of adverse cardiac events in order to assess its prognostic utility.

#### Homoarginine in different patient groups

Our study demonstrates that hArg levels differ significantly between healthy controls and CHD patients, in particular those with univentricular physiology after Fontan palliation and unrepaired cyanotic malformations or Eisenmenger physiology with the latter showing significantly decreased hArg concentrations (Fig 1). This result is not surprising since endothelial dysfunction is frequently present in these subsets of CHD patients. Endothelial dysfunction also plays an important role in the mechanism of a failing Fontan circuit due to endothelin mediated elevation of pulmonary vascular resistance [23–25] or in patients with cyanotic CHD via downregulation of NO synthase in cardiomyocytes resulting in reduced production/ release of NO [26]. If hArg also plays a specific role in this setting remains unclear, however, it is known to be involved in NO metabolism and positively related to endothelial function [10,11].

In our study population, lower hArg levels were also found in patients with adverse cardiac events as compared to those without any cardiac event (Table 1). Moreover, hArg levels decreased significantly with the severity of adverse cardiac events (Fig 2) with the lowest levels found in patients prior to death or overt heart failure indicating the worst scenario of adverse cardiac events. Also, hArg levels were lower in patients with recurrent supraventricular arrhythmias/ permanent atrial fibrillation reflecting altered or even worsening systemic ventricular function and disease progression. It is of note that most of the patients (60%) exhibiting adverse cardiac events in our study had a single ventricle physiology after Fontan palliation or Eisenmenger physiology/ unrepaired cyanotic CHD indicating that the presence of endothelial dysfunction may contribute to the occurrence of adverse cardiac events aside from the thromboembolic complications typically known in these patients [27,28]. The rather low percentage of adverse cardiac events in the other subsets of CHD patients (i.e. patients with repaired CRHD or SRV) may be due to the fact that the majority of these patients were in a good clinical status at the time of enrolment and also remained clinically stable during the follow-up period. This might also be the reason why hArg levels in these subsets of patients did not differ significantly to healthy controls (Fig 1).

#### Association of hArg with other variables

hArg levels were only weakly related to NT-proBNP levels reflecting that both biomarkers represent different pathophysiological pathways. While a strong correlation was found between NT-proBNP levels and measures of systolic ventricular function, hArg levels were not related to these measures. Moreover, hArg showed a rather weak correlation to NYHA functional class. These results are in accordance with a previous study in patients with chronic left heart failure revealing hArg as an independent marker from NT-proBNP and NYHA stages [15].

However, a significant association of hArg with transcutaneous oxygen saturation was found which is often reduced in patients after Fontan palliation or with Eisenmenger physiology/ unrepaired cyanotic CHD. It is known from previous studies that chronic hypoxia leads to alterations in endothelial function [26,29] thus supporting the hypothesis of hArg being involved in NO metabolism. In addition, the enzyme L-arginine:glycine amidinotransferase, which is the major hArg producer, has been shown to be linked to local creatine phosphate synthesis [30]. Thus, one could hypothesize that there is some association between hArg, L-arginine:glycine amidinotransferase and tissue hypoxia as the latter doubtlessly impacts on local energy metabolism.

#### Prediction of death and overt heart failure

According to ROC analysis, the 3 most significant predictors for the occurrence of overt heart failure or death due to progressive heart failure were found to be NYHA class, hArg and NT-proBNP levels with a slightly higher AUC for hArg than for NT-proBNP (Fig 4). These findings demonstrate that NYHA class still is a strong and reliable predictor for overt heart failure despite its subjective aspect of classification but that hArg levels may also predict severe adverse cardiac events and especially the occurrence of overt heart failure as reliably as or even better than NT-proBNP levels. Moreover, repeated measurements (although small in number) demonstrated significantly declining hArg levels prior to the occurrence of overt heart failure or death due to progressive heart failure indicating that serial measurements of hArg levels may identify patients at risk for these severe adverse cardiac events. Furthermore, a significant rise of NT-proBNP levels was observed in some study patients without acute or subsequent deterioration of their clinical status or cardiac function whereas hArg levels remained stable in this setting. Thus, hArg levels seem to be more stable in the long run than NT-proBNP levels what is not surprising because hArg levels do not reflect rapid load-dependent cardiac changes but rather long-standing metabolic alterations. All these results indicate that hArg levels might also be of prognostic value in patients with complex CHD.

## Conclusions

In patients with complex CHD, hArg levels can predict adverse cardiac events as reliably as or even better than NT-proBNP levels and thus might also be of prognostic value in this subset of patients. In contrast to NT-proBNP, hArg levels seem to reflect long-standing metabolic alterations rather than rapid load-dependent cardiac changes.

## Supporting information

S1 Dataset Raw data of patients and controls. (PDF)
