# Supplementary material for: Homoarginine—A prognostic indicator in adolescents and adults with complex congenital heart disease?
Source: PLoS One. 2017 Sep 8;12(9):e0184333. doi: 10.1371/journal.pone.0184333 (PMC5590899; doi:10.1371/journal.pone.0184333)
Supplement: S1 Dataset — (PDF) [file pone.0184333.s001.pdf]

PID	Age at enrollment	AHF group	Gender	Transcutaneous oxygen saturation	Systolic blood pressure
Pat1	15	FONT	female	94	109
Pat2	15	FONT	male	94	98
Pat3	16	FONT	male	95	115
Pat4	11	FONT	male	91	111
Pat5	25	FONT	male	96	109
Pat6	11	FONT	female	91	91
Pat7	11	FONT	male	89	76
Pat8	19	FONT	male	95	146
Pat9	24	FONT	male	92	117
Pat10	22	FONT	female	92	112
Pat11	22	FONT	male	96	128
Pat12	11	FONT	female	89	90
Pat13	19	FONT	female	95	121
Pat14	18	FONT	female	96	126
Pat15	18	FONT	female	95	114
Pat16	17	FONT	male	92	129
Pat17	12	FONT	male	92	121
Pat18	13	FONT	female	91	104
Pat19	16	FONT	female	96	117
Pat20	20	FONT	male	97	99
Pat21	27	FONT	female	90	105
Pat22	18	FONT	male	99	135
Pat23	21	FONT	female	97	121
Pat24	17	FONT	male	95	120
Pat25	11	FONT	male	94	100
Pat26	18	FONT	female	97	106
Pat27	11	FONT	female	94	95
Pat28	36	FONT	female	96	143
Pat29	14	FONT	male	91	125
Pat30	14	FONT	male	92	102
Pat31	14	FONT	male	85	100
Pat32	17	FONT	female	93	120
Pat33	32	FONT	male	92	115
Pat34	14	FONT	female	95	87
Pat35	16	FONT	male	95	132
Pat36	32	FONT	male	94	135
Pat37	18	FONT	male	98	145
Pat38	26	FONT	male	95	125
Pat39	30	FONT	female	84	120
Pat40	45	FONT	male	87	132
Pat41	11	FONT	male	91	98
Pat42	24	FONT	female	92	132
Pat43	13	FONT	male	84	126
Pat44	26	SRV	male	99	118
Pat45	13	SRV	male	98	115

Pat46	23	SRV	male	96	124
Pat47	28	SRV	male	98	130
Pat48	27	SRV	female	98	127
Pat49	28	SRV	female	96	126
Pat50	42	SRV	male	95	115
Pat51	25	SRV	male	96	111
Pat52	21	SRV	female	98	148
Pat53	26	SRV	male	97	136
Pat54	27	SRV	male	95	138
Pat55	21	SRV	male	97	128
Pat56	33	SRV	male	97	141
Pat57	27	SRV	male	99	114
Pat58	29	SRV	male	98	116
Pat59	22	SRV	female	99	122
Pat60	31	SRV	male	98	127
Pat61	48	SRV	female	96	125
Pat62	39	SRV	female	99	130
Pat63	33	SRV	male	98	128
Pat64	24	SRV	male	97	138
Pat65	23	SRV	female	99	110
Pat66	30	SRV	female	98	118
Pat67	21	SRV	male	96	126
Pat68	38	SRV	female	98	101
Pat69	29	SRV	female	98	136
Pat70	26	SRV	male	97	134
Pat71	59	SRV	female	98	129
Pat72	34	SRV	male	95	109
Pat73	24	SRV	male	96	109
Pat74	57	SRV	male	96	142
Pat75	35	SRV	male	98	126
Pat76	61	SRV	male	96	163
Pat77	54	SRV	male	97	118
Pat78	34	SRV	female	98	139
Pat79	18	CRHD	male	96	131
Pat80	13	CRHD	male	97	131
Pat81	26	CRHD	female	96	118
Pat82	15	CRHD	male	100	113
Pat83	18	CRHD	male	99	105
Pat84	73	CRHD	female	96	162
Pat85	41	CRHD	male	95	128
Pat86	36	CRHD	male	96	113
Pat87	61	CRHD	female	95	115
Pat88	47	CRHD	female	99	107
Pat89	54	CRHD	female	96	150
Pat90	23	CRHD	female	100	126
Pat91	27	CRHD	female	98	99

Pat92	24	CRHD	male	98	132
Pat93	24	CRHD	male	97	126
Pat94	23	CRHD	female	98	115
Pat95	33	CRHD	female	98	109
Pat96	27	CRHD	female	100	121
Pat97	30	CRHD	female	100	96
Pat98	18	CRHD	female	97	117
Pat99	34	CRHD	male	97	140
Pat100	21	CRHD	male	99	139
Pat101	39	CRHD	male	97	136
Pat102	25	CRHD	male	94	123
Pat103	16	CRHD	female	95	115
Pat104	22	CRHD	female	99	122
Pat105	18	CRHD	female	96	99
Pat106	20	CRHD	male	97	112
Pat107	27	CRHD	male	99	132
Pat108	48	CRHD	female	99	115
Pat109	33	CRHD	male	99	149
Pat110	31	CRHD	male	99	100
Pat111	27	CRHD	female	96	125
Pat112	36	CRHD	female	98	112
Pat113	40	CRHD	female	96	119
Pat114	28	CRHD	male	95	137
Pat115	46	CRHD	male	97	113
Pat116	33	CRHD	male	98	126
Pat117	39	CRHD	female	98	119
Pat118	22	CRHD	female	98	115
Pat119	19	CRHD	female	98	123
Pat120	15	CRHD	female	95	125
Pat121	41	CRHD	female	99	130
Pat122	23	CRHD	male	96	137
Pat123	20	CRHD	male	100	133
Pat124	25	CRHD	female	98	129
Pat125	20	CRHD	female	99	123
Pat126	29	CRHD	female	96	110
Pat127	18	CRHD	male	100	113
Pat128	48	EIS	female	91	130
Pat129	39	EIS	female	73	148
Pat130	41	EIS	female	74	117
Pat131	33	EIS	male	94	134
Pat132	40	EIS	female	86	120
Pat133	30	EIS	male	76	122
Pat134	34	EIS	male	84	116
Pat135	21	EIS	male	96	119
Pat136	52	EIS	female	72	119
Pat 137	22	EIS	male	88	125

Pat138	34	EIS	male	86	155
Pat139	44	EIS	female	76	126
Pat140	23	EIS	male	94	136
Pat141	40	EIS	female	91	112
Pat142	29	EIS	male	70	123
Pat143	23	EIS	male	76	110
CON1	14				
CON2	20				
CON3	18				
CON4	17				
CON5	52				
CON6	14				
CON7	18				
CON8	12				
CON9	15				
CON10	49				
CON11	48				
CON12	22				
CON13	45				
CON14	54				
CON15	32				
CON16	24				
CON17	42				
CON18	24				
CON19	24				
CON20	25				
CON21	27				
CON22	27				
CON23	33				
CON24	61				
CON25	60				
CON26	24				

Diastolic blood pressure	NYHA functional class	Number of adverse cardiac events	Classification of adverse cardiac events
61	1	0	
60	2	0	
80	1	0	
59	1	0	
58	2	0	
56	2	0	
51	4	1	Death due to progressive heart failure
65	1	0	
71	1	0	
70	2	0	
79	2	0	
60	3	1	Overt heart failure
77	1	0	
78	1	0	
62	1	0	
78	1	1	Sporadic SVT
62	1	0	
61	2	0	
72	1	0	
58	2	0	
60	3	1	Overt heart failure
83	1	1	Sporadic SVT
72	1	0	
70	1	0	
60	2	0	
67	2	0	
59	2	0	
85	2	0	
56	1	0	
57	1	0	
59	1	0	
65	2	1	Overt heart failure
70	1	1	Sporadic SVT
62	2	1	Overt heart failure
66	1	0	
63	3	1	Overt heart failure
50	1	0	
74	1	0	
60	2	1	Overt heart failure
69	3	1	recurrent SVT/AF
65	2	1	Overt heart failure
76	2	0	
73	1	0	
68	2	0	
65	1	0	

72	1	0	
76	2	0	
80	1	0	
82	1	0	
70	1	1	Sporadic SVT
61	3	1	Overt heart failure
97	1	0	
81	1	0	
80	1	0	
78	1	0	
86	1	0	
69	1	0	
69	1	1	Death due to sudden cardiac death
71	1	0	
75	1	1	Sporadic SVT
80	1	1	recurrent SVT/AF
76	2	0	
66	2	0	
79	1	1	Sporadic SVT
77	1	0	
70	2	0	
77	1	0	
62	2	0	
91	1	0	
80	1	0	
79	2	0	
76	2	1	Overt heart failure
69	1	0	
80	3	1	recurrent SVT/AF
57	2	0	
88	2	0	
70	3	0	
75	1	0	
79	2	0	
56	2	1	recurrent SVT/AF
76	2	0	
62	1	0	
61	2	0	
64	2	1	recurrent SVT/AF
76	2	0	
74	1	0	
56	3	2	:current SVT/AF + Death due to progressive heart failu
74	3	1	Overt heart failure
80	2	0	
81	1	0	
68	1	0	

70	1	0	
88	1	0	
77	1	0	
62	1	0	
66	1	0	
56	1	0	
61	1	0	
76	1	0	
70	1	0	
88	1	0	
64	1	0	
71	1	0	
76	1	0	
64	1	0	
59	1	0	
73	1	0	
68	1	0	
83	1	0	
60	1	0	
75	1	0	
61	1	0	
74	1	0	
61	1	0	
64	2	0	
83	1	0	
75	1	0	
71	1	0	
70	1	0	
75	1	0	
75	1	0	
78	1	0	
72	1	0	
76	1	0	
59	1	0	
69	2	0	
71	1	0	
70	3	0	
74	3	1	Overt heart failure
75	3	2	recurrent SVT/AF + Overt heart failure
88	2	0	
65	2	0	
82	3	1	Overt heart failure
68	2	0	
68	2	0	
66	2	0	
72	2	0	

92	2	0	
84	2	0	
80	3	1	Overt heart failure
79	3	0	
79	3	2	recurrent SVT/AF + Overt heart failure
57	3	1	Death due to progressive heart failure

NT-proBNP levels	Homoarginine levels	Gamma GT levels	Albumin levels	Creatinine	Estimated GFR
67,9	1,6	78	48	0,6	126,8
69,1	0,78	49	46	0,8	124,1
35,8	2,03	138	51	0,67	92
53,4	1,17	43	46	0,6	
425,1	1	169	43	1	88,9
131,7	1,42	26	47	0,6	
9408	0,54	64	43	0,4	
267,3	1,17	81	51	1,13	93,3
83,5	1,03	90	50	0,94	113,2
136,7	1,6	120	45	0,7	105,2
60,6	1,74	32	49	1	99,6
473	0,67	72	37	0,5	
57,8	1,43	80	51	1	69,5
940,8	1,3	67	44	0,8	97
85,8	2,15	80	48	0,7	104,8
56,8	1,32	69	44	0,75	118
255,8	0,83	98	44	0,5	
37,3	1,32	45	46	0,6	
100,7	1,78	24	45	0,8	79,6
132,7	1,93	113	51	0,7	146,1
631	0,76	247	38	0,49	133,5
73,4	1,34	66	50	0,7	118,4
182,6	0,75	91	48	0,8	88,8
19	0,95	42	43	0,93	104,5
77,7	1,23	34	49	0,5	
45	1,64	124	45	0,97	74,4
74,8	1,92	25	50	0,5	
110,3	3,85	218	42	0,7	101,5
119,8	1,27	26	46	0,86	
102,2	1,34	64	45	0,39	
272,1	0,95	77	48	0,6	
115,9	0,79	81	53	0,8	
139,1	1,21	58	45	0,88	114
386,5	1,07	47	19	0,37	
66	1,44	63	43	0,88	
1111	0,91	170	52	1,28	73,6
77,4	1,92	57	46	0,89	111,5
217,3	0,84	68	48	0,9	117,3
641,1	0,43	97	41	0,58	122
1787	1,41	72	50	0,92	93,5
133,6	0,6	30	42	0,37	
194,7	2,04	43	47	0,5	135,2
39,6	0,63	67	41	0,45	
855,6	1,29	21	49	0,77	124,8
122,1	2,43	26	47	0,77	

980,4	1,16	44	46	1,11	92,6
747,1	1,37	120	43	1,08	92,8
214	3,03	29	49	0,8	90,3
164,5	1,92	15	50	0,5	137
928,2	1,62	74	47	0,95	98,6
932,7	1,21	118	52	0,72	133,6
92,6	1,29	44	46	0,8	98
74,5	2,41	72	52	0,93	97,7
96,5	2,51	41	44	1	94,4
122,3	3,24	22	45	1,16	79,8
431,6	1,98	60	48	0,88	112,6
88,7	1,6	34	46	0,81	114,2
208,7	4,29	18	50	0,96	91,4
188	1,09	9	44	0,72	119,8
222,1	1,29	60	45	1,1	80,5
292,7	0,5	17	46	1,02	65
738,8	2,96	34	46	0,64	103
510,8	1,76	25	49	0,84	105
586,3	1,53	91	49	0,96	110,3
78,8	2,59	24	46	0,92	75,9
212	0,93	24	51	0,97	67,7
42,4	1,33	23	45	0,86	112,4
690,8	1,36	26	46	0,73	89,5
421,2	1,6	41	48	0,76	90,1
67,7	3,26	18	48	0,94	96,5
522,7	2,14	27	47	0,84	76,5
901,5	1,25	71	45	1,17	80,9
279,5	1,52	34	47	1,13	90,7
660,8	2,98	137	48	0,83	97,7
746,4	1,4	30	45	0,86	112,7
334,9	3,23	60	42	1,09	76,2
286,8	1,98	62	42	0,89	97,1
501,1	2,1	24	47	0,59	120,3
42,1	2,22	36	51	1	948
354,4	1,5	48	50	0,5	126
1531	1,16	85	48	0,7	119,9
57,6	2,13	18	49	0,76	128,6
402,9	3,04	63	44	0,8	100,9
1351	0,78	126	51	0,7	
1054	1,45	54	44	0,94	88,7
65,1	1,68	19	43	0,76	116,2
1318	1,03	93	28	1,25	37,3
2765	0,89	60	44	2,18	26,1
624,1	2,49	79	45	0,62	102,3
74,1	2,53	12	47	0,76	111,3
145	2,04	10	47	0,63	123,5

166,8	1,54	16	49	0,88	120,8
113	2,02	34	44	0,94	113,3
192,4	3,11	28	48	0,72	119,1
205,5	1,26	27	46	0,74	106,9
145,2	1,39	14	44	0,64	123,1
147,1	1,75	85	42	0,61	121,9
136,8	1,13	25	49	0,67	120,1
62,5	2,22	24	49	1,08	89,3
33,5	1,83	22	47	1,21	85,1
258,5	1,63	70	48	0,81	111,5
266,3	1,62	18	51	0,97	107,9
41,1	1,14	13	52	0,84	110,9
48,8	3,1	22	49	0,86	95,8
139	1,85	7	47	0,84	101,8
57,2	1,76	57	53	0,94	116,2
68,6	0,98	35	48	0,77	124,5
94,4	2,29	11	48	0,68	103,8
55,7	2,26	30	46	0,86	113,8
151,7	2,77	22	47	1,05	93,8
162,8	1,12	34	49	0,8	101,2
91,9	0,88	74	52	0,81	93,2
334,3	1,9	41	44	0,76	98,3
50,8	1,55	30	49	0,97	106,3
200,7	1,82	11	44	0,93	98,2
164,7	1,81	16	48	1,03	94,6
71,7	1,54	13	46	0,77	97,5
204	1,76	45	47	0,61	129
96,5	1,6	43	47	0,72	121,5
85	2,2	19	49	0,58	
137,2	2,13	13	51	0,76	97,4
38,4	1,95	25	52	0,82	124
26,7	1,34	21	56	0,77	130,5
187,4	2,14	57	42	0,68	121,3
92,9	2,66	16	48	0,66	127,3
55,2	3,04	19	44	0,9	86,4
51,8	1,36	34	46	0,76	132,9
1083	0,46	33	53	1,1	55,4
7771	1,5	58	38	0,84	88,2
722,2	1,02	21	42	0,61	112,8
259	1,37	44	38	0,91	110,4
579	1,32	35	49	1,04	58,2
4056	0,78	17	37	1,1	89,3
413,9	0,94	38	51	0,87	112,7
28,5	1,75	21	50	0,66	152,2
88,1	0,81	19	44	1,28	47,8
631,2	1,84	18	48	1,07	97,7

310,4	1,1	37	48	0,9	93,2
652,1	0,92	20	42	0,95	73,1
984,4	0,79	198	42	1,1	94,9
1318	1,77	40	47	0,8	80,5
2093	0,62	251	40	1,1	88,7
347,4	0,96	72	47	0,8	126,2
	1,72				
	1,46				
	1,89				
	1,97				
	1,24				
	1,59				
	1,61				
	2,8				
	1,39				
	1,68				
	1,72				
	2,95				
	1,01				
	1,33				
	3,17				
	1,51				
	1,96				
	1,96				
	2,24				
	1,67				
	1,34				
	2,27				
	1,06				
	1,39				
	4,63				
	2,03				

Endsystolic volume of systemic ventricle	Enddiastolic volume of systemic ventricle	Ejection fraction of systemic ventricle
41,74	82,36	49,3
37,32	92,6	60,2
75,89	154,88	51
39,83	116,12	65,7
87,1	159,84	45,5
29,43	84,61	65,2
139,3	183,2	24
71,96	155,98	53,9
47,01	107,78	56,4
18,65	60,9	69,4
31,08	87,99	64,7
23,35	42,9	45,6
145,38	252,68	45
33,04	85,17	61,2
28,77	57,1	49,6
48,07	123,28	61
61,1	149,85	59,2
37,3	87,01	57,2
38,41	98,82	61,1
125,95	212,86	40,8
28,98	74,69	61,2
67,4	171,3	60,6
76,2	163,6	53,4
26,02	74,4	65
58,51	105,12	63,8
60,89	135	54,9
37,53	68,62	61
95,06	167,98	43,4
29,3	80,88	63,8
43,37	110,57	60,8
44,67	92,1	51,5
38,83	99,07	60,8
21,57	50,1	57
90,3	137,4	34,5
35,95	74,9	52
74,4	167,22	55,5
75,87	171,63	55,8
89,71	177,03	49,3
48,82	96,92	49,6
76,99	105,68	27,2
27,53	97,1	71,6
61	163	63
33,3	96,2	65,4
89,65	130,01	31
47,93	92,33	48,1

154,74	193,2	33,7
76,66	126,08	39,2
30,8	53,7	43
54,52	85,5	47
95,29	125,5	24,1
241,28	290,2	16,9
23	49,4	53
48,73	78,1	41,2
73	165	56
61,13	107,94	43,4
38,98	78,81	50,5
58,43	116,2	49,7
86,8	152,8	43
42,64	80,84	47,3
35,5	67,6	48
47,89	84,46	43
56,37	90,88	38
37,52	74,54	49,7
93,91	162,31	42,1
20,9	53,56	61
36,85	69,05	46,6
40	62	36
37,18	61,81	39,8
36,43	64,26	43,3
67,16	120,28	40,5
90,13	128,76	30
85,32	120,86	29,4
65,95	111,81	41
71,66	97,73	26,7
67,1	108,2	38
70,43	112,1	37,2
35,94	77,22	53,5
44,43	93,31	52,4
47,4	97,02	51,1
17,8	57,83	70,5
69,82	129,35	46
42,03	97,99	57,1
38,98	65,93	40,9
43,82	81,57	46,3
52,85	102,08	48,2
41,65	101,82	59,1
39,51	62,12	36,4
107,57	136,61	21,3
41,78	115,63	63,9
37,67	87,44	56,9
36,77	78,47	51,1

56,69	117,72	51,8
71,06	140,29	49,4
32,75	84,91	51,7
58,14	132,87	56,2
54,6	108,25	49,5
45,98	88,7	48,2
26,74	67,31	62,3
56,1	153,63	63,5
61,42	149,98	59,1
70,1	149,4	53,1
67,61	160,65	57,9
33,85	95,52	52,8
30,81	97,29	68,3
14,26	47,81	70,2
40,4	82,4	58,9
40,72	83,02	50,9
35,28	83,33	57,7
37,27	105,04	64,5
41,03	68,03	39,7
36,89	79,48	53,6
35,91	82,16	56,3
54,05	94,9	43
67,57	207,64	67,5
60,93	109,63	44,4
62,72	113,47	45
53,57	119,02	55
36,43	93,5	61
39,31	119,66	67,2
45,69	122,41	62,7
56,37	134,34	58
58,63	135,78	56,8
61,67	144,59	58,9
32,87	84,15	60,9
28,64	89,75	68,1
47,2	127,03	62,8
18,22	40,13	55,2
26,3	55,16	52,3
104	156	33
85	153	44,4
18,12	41,65	56,5
81	152	37
80,11	127,25	37,1
115,96	198,69	41,6
30,33	86,25	64,8
19,6	40,73	51,7
31,52	71,26	55,8

64	116	46,6
32,15	88,85	51,5
71,54	122,1	41,4
24	64	62,5
174,5	241,37	27,7
60,97	125,1	51,3

VTI above aortic valve

VTI above aortic valve
28,3
29,6
25,2
31,8
38,1
20,4
12,8
21,1
24,6
21,7
34,8
19,6
16,9
26,6
17,5
30,4
29
19
28,8
19,4
24,2
26,7
25,1
24,4
20,3
26,9
22,4
28,9
28
23,7
17,9
28,4
25,4
35,2
31
22,8
20,5
25,4
21,9
22,2
16,9
22,2
23,5
24,7
27,4

19,3
17,5
24,6
26,4
20,3
17,1
38,5
21,7
27,3
29
26,7
26,4
26,4
26,7
27,6
21,4
27,3
19,6
16,3
23
23,8
24,5
22,1
22,5
23,8
19,7
15,1
27,8
16,1
22,6
24,1
23,7
25,6
20,7
31,3
15,8
25
31,1
23
24,3
24,2
26,3
13,2
30,3
26,7
22,5

17,1
31,9
21,6
31,9
27,8
28,8
22,5
21,9
24,1
22
26,5
29,3
24,5
21,8
21,6
22,4
28,2
29,8
21,1
27,4
29,9
21,8
24,8
16,9
19,4
22,1
17,2
26,1
28,1
20,7
24
23,3
25
29,3
26,4
24,2
19,4
22,4
16
21,6
33,2
14,6
19,8
23,7
22,9
37,1

28,2
24,4
18,1
21,1
17,9
12,5
